# Association Between Social Cognition Changes and Resting State Functional Connectivity in Frontotemporal Dementia, Alzheimer’s Disease, Parkinson’s Disease, and Healthy Controls

**DOI:** 10.3389/fnins.2019.01259

**Published:** 2019-11-22

**Authors:** Namita Multani, Foad Taghdiri, Cassandra J. Anor, Brenda Varriano, Karen Misquitta, David F. Tang-Wai, Ron Keren, Susan Fox, Anthony E. Lang, Anne Catherine Vijverman, Connie Marras, Maria Carmela Tartaglia

**Affiliations:** ^1^Tanz Centre for Research in Neurodegenerative Diseases, University of Toronto, Toronto, ON, Canada; ^2^Memory Clinic, Toronto Western Hospital, University Health Network, Toronto, ON, Canada; ^3^The Edmond J. Safra Program for Parkinson Disease, Movement Disorder Clinic, Toronto Western Hospital, University Health Network, Toronto, ON, Canada

**Keywords:** neurodegeneration, social cognition, resting-state fMRI, neuroimage analysis, functional connectivity

## Abstract

**Objective:**

To determine the relationship between alterations in resting state functional connectivity and social cognition dysfunction among patients with frontotemporal dementia (FTD), Alzheimer’s disease (AD), Parkinson’s disease (PD), and healthy controls (HC).

**Methods:**

Fifty-seven participants (FTD = 10, AD = 18, PD = 19, and HC = 10) underwent structural and functional imaging and completed the Awareness of Social Inference Test-Emotion Evaluation Test (TASIT-EET), Behavioral Inhibition System/Behavioral Activation System (BIS/BAS) scale, Revised Self-Monitoring Scale (RSMS), Interpersonal Reactivity Index (IRI), and Social Norms Questionnaire (SNQ). A multi-variate pattern analysis (MVPA) was carried out to determine activation differences between the groups. The clusters from the MVPA were used as seeds for the ROI-to-voxel analysis. Relationship between social cognition deficits and uncinate integrity was also investigated.

**Results:**

BOLD signal activation differed among the four groups of AD, PD, FTD, and HC in the left inferior temporal gyrus-anterior division [L-ITG (ant)], right central opercular cortex (R-COp), right supramarginal gyrus, posterior division (R-SMG, post), right angular gyrus (R-AG), and R-ITG. The BOLD co-activation of the L-ITG (ant) with bilateral frontal pole (FP) and paracingulate gyrus was positively associated with IRI-perspective taking (PT) (*r* = 0.38, *p* = 0.007), SNQ total (*r* = 0.37, *p* = 0.009), and TASIT-EET (*r* = 0.47, *p* < 0.001).

**Conclusion:**

Patients with neurodegenerative diseases showed alterations in connectivity in brain regions important for social cognition compared with HCs. Functional connectivity correlated with performance on social cognition tasks and alterations could be responsible for some of the social cognition deficits observed in all neurodegenerative diseases.

## Introduction

Neurodegenerative diseases consist of a heterogeneous group of conditions, including frontotemporal dementia (FTD), Alzheimer’s disease (AD), and Parkinson’s disease (PD), that present with different clinical syndromes determined by the different brain areas and circuits most often affected. The focus of research in neurodegenerative disease has been the cognitive domains of memory, language, executive, and visuospatial function. Social cognition comprises many psychological processes including perceiving and recognizing social and emotional signals, evaluating the personal emotional relevance of everyday information, maintaining and accessing common social knowledge, processing information about beliefs and intentions, and generating and selecting behavioral responses that enable an individual to participate in social interactions. There is growing awareness that social cognitive deficits, including disturbances of emotion recognition, occur in the different neurodegenerative diseases (Snowden et al., 2003; Shany-Ur and Rankin, 2011; Sollberger et al., 2014; Poveda et al., 2017).

Frontotemporal dementia comprises a number of clinical syndromes involving behavior, language, and motor dysfunction. The main syndromes encompassed by the clinical term FTD are behavioral variant (bvFTD), non-fluent variant primary progressive aphasia (nfvPPA), and semantic variant primary progressive aphasia (svPPA). The clinical expression of these syndromes is determined by the selective injury of specific areas of the brain, which leads to the diverse signs and symptoms. Dramatic personality and behavioral changes with apathy, disinhibition, prominent loss of social cognition, lack of empathy, and inability to decipher other’s emotions, are hallmarks of bvFTD (Gustafson, 1987; Neary et al., 1988, 1998). Social cognition deficits are early signs of bvFTD. There are a number of studies that have reported various social cognitive abnormalities in bvFTD patients, including abnormalities in Theory of Mind (ToM) detection of gaze direction, and recognition of facial and/or prosodic emotional expressions, in particular negative emotions such as fear and anger (Gregory et al., 2002; Keane et al., 2002; Rosen et al., 2004; Lavenu and Pasquier, 2005; Diehl-Schmid et al., 2007; Eslinger et al., 2007; Kessels et al., 2007; Werner et al., 2007; Bediou et al., 2009). There is evidence that bvFTD patients have difficulty identifying social concepts, judging appropriate actions in social dilemmas, recognizing sarcasm, and differentiating minor social transgressions from serious moral violations (Mendez et al., 2005; Lough et al., 2006; Eslinger et al., 2007; Grossman et al., 2010; Shany-Ur et al., 2012). Although svPPA and nfvPPA are primarily identified as language disorders, social cognition can also be affected (Neary et al., 1998; Hodges and Miller, 2001; Multani et al., 2017) and loss of emotion detection and decreased empathy has been reported in svPPA and nvPPA (Multani et al., 2017).

Individuals diagnosed with AD often display episodic memory dysfunction, accompanied by neuropathologic, metabolic, and functional connectivity changes within the medial temporal lobe, posterior cingulate cortex, precuneus, and lateral temporoparietal areas, suggesting impairment throughout a posterior episodic memory network (Greicius et al., 2004; Buckner et al., 2005). In AD, social cognition has received less attention but there is increasing evidence that patients with AD have impaired ToM (Koff et al., 2004) as well as decreased ability to recognize emotions (Hargrave et al., 2002; Burnham and Hogervorst, 2004; Kohler et al., 2005; Bediou et al., 2009; Martinez et al., 2018). Studies have also revealed that the social cognition deficits are not necessarily correlated with cognitive deficits (Hargrave et al., 2002; Bediou et al., 2009) although there is controversy over this (Burnham and Hogervorst, 2004; Spoletini et al., 2008). One notable study evaluated facial emotion expression recognition and noted that the worst performers were not those with the worst cognitive scores (Luzzi et al., 2007). The default mode network, a prime target in AD, has been implicated in social cognition so it shouldn’t be surprising that multiple studies are demonstrating social cognitive deficits in AD (Torralva et al., 2000; Bediou et al., 2009; Zhou et al., 2010).

Although PD is known for its motor impairments, non-motor deficits including social cognitive deficits are likely due to disruption of fronto-striatal circuits due to impaired dopamine release (Zgaljardic et al., 2006; Sawamoto et al., 2008; Skuse and Gallagher, 2009; Bodden et al., 2010). Several studies have reported that PD patients have an impaired ability to recognize facial, and to a lesser extent prosodic, expressions of emotion, particularly disgust, fear, and anger (Kan et al., 2002; Mengelberg and Siegert, 2003; Pell and Leonard, 2003, 2005; Yip et al., 2003; Dujardin et al., 2004; Mimura et al., 2006; Kawamura and Koyama, 2007; Benuzzi et al., 2008; Assogna et al., 2010; Martinez et al., 2018), but this has not been a consistent finding (Biseul et al., 2005; Pell and Leonard, 2005). Although the neural basis of these deficits is not fully understood, various neural substrates have been implicated in emotion recognition including amygdala, orbitofrontal cortex (OFC), insula, and basal ganglia (BG) (Adolphs, 2002; Adolphs et al., 2003; Hornak et al., 2003; Krolak-Salmon et al., 2003; Sprengelmeyer et al., 2003; Yoshimura et al., 2005; Fusar-Poli et al., 2009; Baggio et al., 2012). The BG, a major site of dysfunction in PD because of its loss of dopaminergic innervation, has been implicated in facial emotion recognition (Cancelliere and Kertesz, 1990; Adolphs et al., 2002) as well as in emotion recognition from prosodic cues, where it acts in conjunction with the right frontoparietal cortex and potentially the amygdala (Cancelliere and Kertesz, 1990; Starkstein et al., 1994; Adolphs et al., 2002). A few studies have demonstrated emotion recognition deficits and bilateral orbitofrontal and right amygdala atrophy in patients with PD (Ibarretxe-Bilbao et al., 2009). PD patients have also shown impaired TOM although it’s felt that cognition may be contributing to that impairment (Saltzman et al., 2000; Yu et al., 2012).

These social cognitive changes that often include changes in personality are a source of distress for caregivers (Martinez et al., 2018) and their neuroanatomical correlates require further investigation. Moreover, in addition to gray matter atrophy, there is increasing evidence for network dysfunction and functional connectivity alteration in neurodegenerative diseases (Seeley et al., 2009). FTD encompasses behavioral and language syndromes including behavioral variant FTD (bvFTD), semantic variant PPA, and non-fluent variant PPA. The most frequently studied FTD group with respect to networks is bvFTD and alterations in the saliency network is the most consistent finding (Zhou et al., 2010; Day et al., 2013; Farb et al., 2013; Filippi et al., 2013; Caminiti et al., 2015). Some studies have reported an increased default mode network connectivity in FTD (Zhou et al., 2010; Farb et al., 2013; Rytty et al., 2013), but others have reported reductions within the default mode network (Farb et al., 2013; Filippi et al., 2013). Changes in connectivity of executive control network, dorsal attention network, the auditory network (Hafkemeijer et al., 2015), and the frontoparietal and frontotemporal networks including the insular cortex (Farb et al., 2013; Rytty et al., 2013; Sedeño et al., 2016) have also been reported. Comparing patients with bvFTD and AD directly, opposite patterns emerged with increased connectivity of default mode network and disrupted saliency network in bvFTD and the opposite pattern in AD (Zhou et al., 2010).

The default mode network, encompassing the posterior cingulate, precuneus, inferior parietal cortex, OFC, medial prefrontal cortex, ventral anterior cingulate (ACC), left dorsolateral prefrontal cortex, left parahippocampus, inferior temporal cortex, nucleus accumbens, and the midbrain (Raichle et al., 2001; Greicius et al., 2003) are responsible for a baseline state of the brain that represents self-reference, emotional processing, memory, as well as spontaneous cognition and aspects of consciousness (Raichle, 2015). Altered connectivity within the DMN in particular connectivity of the precuneus, posterior cingulate cortex, and the prefrontal cortex have been implicated in AD (He et al., 2007; Sorg et al., 2007; Wang et al., 2007; Gili et al., 2011; Wu et al., 2011; Agosta et al., 2012; Binnewijzend et al., 2012; Filippi et al., 2013; Liu et al., 2013; Weiler et al., 2014; Hafkemeijer et al., 2015; Zhou et al., 2015). Based on the division into anterior and posterior default mode network, it was found that connectivity reductions in the default mode network are mainly found in the posterior default mode network (Koch et al., 2014), but with altered connectivity to the anterior default mode network (Jones et al., 2012).

Altered connectivity affecting motor regions including the supplementary motor area, the premotor area, the primary motor cortex, the cerebellum, BG, and thalamic connectivity have been reported in PD (Wu et al., 2009, 2011; Kwak et al., 2010; Hacker et al., 2012; Skidmore et al., 2013; Luo et al., 2014, 2015; Szewczyk-Krolikowski et al., 2014; Canu et al., 2015; Rolinski et al., 2015). Alterations in connectivity of amygdala to frontal, occipital, and cerebellar locations have also been reported in PD (Hu et al., 2015). Unlike in AD, no clear patterns regarding the default mode network are present with some papers reporting either no (Helmich et al., 2009; Krajcovicova et al., 2012) or only few (Tessitore et al., 2012; Disbrow et al., 2014; Canu et al., 2015) alterations of default mode network connectivity although one paper found rather substantial alterations in PD compared with healthy controls (HCs) (Chen et al., 2015). Increased default mode network connectivity was also found in some papers (Campbell et al., 2015; Gorges et al., 2015). Widespread alterations of networks in PD, within and between networks have been reported as well (Madhyastha et al., 2015; Onu et al., 2015). An investigation of the executive control network did not yield any differences in PD compared to healthy adults (Disbrow et al., 2014), but another study found impaired connectivity between the right central executive network and the salience network in PD (Putcha et al., 2015). Disrupted connectivity in frontal and parietal networks in PD with dementia (Amboni et al., 2015; Borroni et al., 2015), as well as in the dorsal attention network (Baggio et al., 2015) have been reported. Several networks have been identified to play a key role in distinct social cognitive functions. The default mode network, which is altered in AD (Greicius et al., 2004), is hypothesized to be involved in introspection. The salience network, on the other hand, disrupted in bvFTD, plays a key role in directing attention to behaviorally relevant salient information.

In recent years, diffusion tensor imaging (DTI) studies have revealed significant white matter tract abnormalities in addition to the previously known gray matter atrophy in neurodegenerative diseases (Zhang et al., 2009; Caso et al., 2016; Multani et al., 2016; Elahi et al., 2017). DTI allows *in vivo* evaluation of white matter integrity by examining distribution of water molecules within fiber tracts (Ciccarelli et al., 2008). Decreased integrity of the right uncinate fasciculus (UF) has been related to changes in emotion detection and cold-heartedness in language variants of FTD (Multani et al., 2017).

Although certain network alterations have been identified in these neurodegenerative diseases, it is unclear whether social cognition relevant regions have altered functional activity in these diseases. Previous studies highlight network-wide (connectivity between multiple neuroanatomical regions) connectivity dysfunction, which may potentially mask changes in region-to-region (ROI-to-ROI) functional connectivity (connectivity between two specific regions) and its association with social cognition function. Furthermore, ROI-to-ROI functional connectivity analysis can be used in conjunction with DTI (structural) analysis to understand whether the observed behavior or symptom is associated with functional or structural dysfunction between the two regions.

Given the vast number of neuroanatomical regions involved in social cognition, a whole-brain level functional connectivity analysis can provide insight into particular brain regions that are altered in different neurodegenerative diseases. We hypothesized that these whole-brain level disease-specific alterations in functional connectivity could help identify regions that would vary across AD, FTD, and PD, and that these changes would relate to abnormalities in social cognitive function.

## Materials and Methods

### Study Participants

Subjects with neurodegenerative diseases, along with their caregivers were recruited from the Memory Clinic and Movement Disorders Clinic, Toronto Western Hospital. Subjects qualified for the study if they had one of the following diagnosis: (1) AD as outlined by McKhann et al. (2011) (*N* = 18); (2) PD according to the PD Society Brain Bank Clinical Diagnostic Criteria (Hughes et al., 1992) (*N* = 19); (3) ten patients with FTD subtypes (svPPA *N* = 4), nfvPPA (*N* = 2) as outlined by Gorno-Tempini et al. (2011), or bvFTD (*N* = 4) as per Rascovsky et al. (2011) (Gorno-Tempini et al., 2011; Rascovsky et al., 2011); (4) age and sex matched HCs with no history of neurological or psychiatric disorders were also recruited (*N* = 10). Patients were excluded from the study if they had any of the following: presence of other neurological disorder, psychiatric illness, and visual or auditory deficits requiring correction beyond corrective lenses or hearing aids, respectively. The study received ethics approval of the University Health Network Research Ethics Board and all subjects provided written consent.

### Measures

Participants (patients, caregivers, and HCs) completed The Awareness of Social Inference Test-Emotion Evaluation Test (TASIT-EET) to evaluate their ability to recognize emotions (McDonald et al., 2003). Cognition was evaluated using the Toronto Cognitive Assessment (TorCA) in all participants (Freedman et al., 2018) and caregivers were interviewed on the Clinical Dementia Rating (CDR) scale to assess dementia severity in patients (Morris, 1993). Social cognition measures were assessed using the following informant-based questionnaires: (1) Behavioral Inhibition System/Behavioral Activation System (BIS/BAS) scale (Carver and White, 1994) to measure behavioral inhibition (sensitivity to punishment) and behavioral activation (sensitivity to rewards) in individuals, (2) Revised Self-Monitoring Scale (RSMS) (Lennox and Wolfe, 1984) in order to measure the subjects’ awareness of their own social behavior (as assessed by the informant), we obtained informant’s perspective of the subject’s self-concern and self-focus using the Lennox and Wolfe version of the RSMS informant-based reports (Lennox and Wolfe, 1984). (3) Interpersonal Reactivity Index (IRI) (Davis, 1983) to measure empathy, which measures both the cognitive and emotional aspects of empathy, and (4) Social Norms Questionnaire (SNQ) (Rankin, 2008) to assess the subject’s ability to assess social boundaries in the mainstream culture of Canada. For participants with PD, these assessments were all performed in the on drug state. Detailed descriptions of scales are available in Supplementary Material.

### Magnetic Resonance Imaging Acquisition

All structural and resting state scans were performed on a 3 Tesla Magnetic Resonance Imaging (MRI) Scanner (GE Signa HDx, Milwaukee, WI, United States) with a standard 8-channel head coil. High resolution T1-weighted images were obtained using inversion recovery fast spoiled gradient echo (IR-FSPGR), with the following parameters: 176 slices with 1.2 mm thickness; 2.8 ms echo time (TE); 7.0 ms repetition time (TR); 400 ms inversion time (TI); 11° flip angle; 26.0 cm field of view (FOV); 256 × 256 matrix size. T2^∗^-weighted functional data images were acquired in an interleaved order (28–32 slices for the whole brain), using the following parameters: slice thickness = 5 mm with no gap, FOV = 240 mm, TR = 2 s. For each participant, two 3-min scans were acquired. The scans were acquired in an oblique orientation and each slice was perpendicular to the long axis of the hippocampus. DTI scans were acquired in 8 min with 55 directions, using the following parameters: 2D single-shot EPI sequence, 60 contiguous slices, slice thickness = 2 mm, TR = 800 ms, TE = 100 ms, *b* = 1000 s/mm^2^, base matrix = 128 × 128, and FOV = 240 × 256 mm^2^.

### Resting State fMRI Analysis

Resting state fMRI preprocessing and analysis was conducted using the Conn Toolbox 17e^1^ (RRID:SCR_009550) (Whitfield-Gabrieli and Nieto-Castanon, 2012). The preprocessing pipeline for structural and functional images consisted of the following: (1) slice-time correction in ascending order, (2) functional realignment and unwarp, (3) co-registration of functional images to structural images, (4) structural segmentation into gray matter, white matter and cerebrospinal fluid, and normalization to MNI space, (5) functional normalization to MNI space, (6) the Artifact Detection Tools (ARTs) scrubbing method for global signal outlier and motion detection, which were used as first level covariates, and (7) functional smoothing (FWHM = 8 mm).

Since the purpose of the study was to examine differences in functional connectivity and its association with social cognition function, as the first step, an exploratory voxel-to-voxel analysis was conducted to determine differences in peak blood oxygen-level dependent (BOLD) activation between the four groups (i.e., AD, PD, FTD, and HC groups). Once differences in peak activations were established, we then used MVPA at the group level to extract seeds to use for ROI-to-voxel connectivity analysis as the second step. Since there is an intricate relationship between neuroanatomical regions associated with social cognition function, this approach enabled us to only address regions whose functional activity is altered across the AD, FTD, and PD groups. Therefore, the seeds from the MVPA, which were associated with social cognition, were extracted as ROI masks and were applied for ROI-to-voxel analysis to determine differences in activation between the four groups. Lastly, ROI-to-voxel Fisher’s *Z*-transformation scores were extracted to conduct two-tailed partial correlations, while controlling for CDR-SoB and sentence comprehension, with social cognition measures (TASIT-EET, SNQ total, BIS, IRI-EC, IRI-PT, RSMS-EX, RSMS-SP). The ToRCA sentence comprehension score (language) was used as a covariate when conducting partial correlations for TASIT-EET and SNQ total as language comprehension may impede performance.

### Diffusion Tensor Imaging and Structural Connectivity

The fMRIB Software Library (FSL) tools^2^ was used to conduct DTI analysis. The DTI processing, region of interest definition for the UF, and fiber tracking were performed as previously described (Galantucci et al., 2011; Taghdiri et al., 2018). Four measures were obtained for the UF: fractional anisotropy (FA), axial diffusivity (AxD), radial diffusivity (RD), and mean diffusivity (MD).

### Statistical Analysis

Statistical analyses were conducted using SPSS software (SPSS Inc. v. 24). One-way ANOVA was conducted to determine group differences on age, TorCA cumulative percentage, CDR SoB, BIS, BAS-D, BAS-FS, BAS-RR, IRI-PT, IRI-EC, RSMS-EX, RSMS-SP, SNQ total score, and the DTI measures (i.e., FA, AxD, RD, and MD) of right and left UF. Dunnett T3 (Shingala and Rajyaguru, 2015) analysis was carried out as a *post hoc* analysis. One-way ANCOVA was carried out to examine differences on TASIT-EET performance, while controlling for TorCA sentence comprehension.

Since the frontal pole (FP) and the inferior temporal gyrus (ITG) (anterior division) are structurally connected by the UF, we carried out mediation analyses to explore the potential mediation effects of the left UF on the relationship between “Group” (coded as 1 = HC, 2 = PD, 3 = AD, and 4 = FTD) and the functional connectivity between the bilateral FP and the left ITG and post-cingulate gyrus (Model A). The right UF is also known to contribute to social cognition (Oishi et al., 2015), so a similar mediation analysis was conducted to investigate the potential mediation effects of the right UF on the relationship between the “Group” and the functional connectivity between the right ITG and FP (Model B). To do so, we used the SPSS macro PROCESS tool (Hayes, 2013) which is implemented in SPSS version 24. We estimated the total, direct, and indirect effects as well as their associated standard error (SE) and 95% confidence intervals (CIs) (while controlling for age) using the 5000 bootstrap samples (Hayes, 2009, 2013). Mediation ratio was also calculated for each model as the ratio of direct effect to the total effect (Preacher and Kelley, 2011). All mediation analyses adjusted for participants’ age. For each specific effect, if the interval did not contain zero, it was considered statistically significant.

## Results

After correcting for multiple comparisons, a significant difference was found between the four groups for TorCA cumulative percentage, CDR SoB, and IRI-PT (Table 1). In addition, there was a trend toward significance in comparison of the SNQ total score and TASIT-EET between the groups.

**TABLE 1:** Demographics, clinical profiles, and social cognition measures.

	**AD (*N* = 18)**	**PD (*N* = 19)**	**FTD (*N* = 10)**	**HC (*N* = 10)**	***p*-value**
Age (years)	70.56 ± 10.4	70.26 ± 9.1	65.5 ± 9.2	62.5 ± 5.5	0.121
Gender (f/m)	11/7	3/16	2/8	6/4	0.01
TorCA Cum %	59.2 ± 17^a,c^	76.7 ± 13^a,b^	61.1 ± 17^a^	91.8 ± 3^b,c,d^	< 0.001^∗^
CDR SoB	4.5 ± 2^a^	2.9 ± 2^a^	4.0 ± 3^a^	0.0 ± 0^b,c,d^	< 0.001^∗^
TASIT EET	8 ± 2	9 ± 2	7.3 ± 3	11 ± 2	0.008
BIS	18.6 ± 6	20.4 ± 5	16.6 ± 3	17.9 ± 4	0.231
IRI-PT	19.6 ± 6^a^	21.0 ± 7^a^	14.7 ± 9^a^	28.6 ± 4^b,c,d^	0.001^∗^
IRI-EC	25.0 ± 6	26.6 ± 7	21.2 ± 8	28.1 ± 5	0.148
RSMS-EX	18.4 ± 7	18.0 ± 6	10.9 ± 8	19.9 ± 7	0.028
RSMS-SP	22.0 ± 5	20.6 ± 8	14.4 ± 10	24.4 ± 7	0.033
SNQ total	17.1 ± 2	18.2 ± 2	15.1 ± 3	19.1 ± 2	0.008

*^∗^Bonferroni correction for multiple comparison, significant if *p* < 0.006. ^*a*^*p* < 0.05 compared to HC. ^*b*^*p* < 0.05 compared to AD. ^*c*^*p* < 0.05 compared to PD. ^*d*^*p* < 0.05 compared to FTD.*

### Voxel-to-Voxel Analysis

**FIGURE 1:**
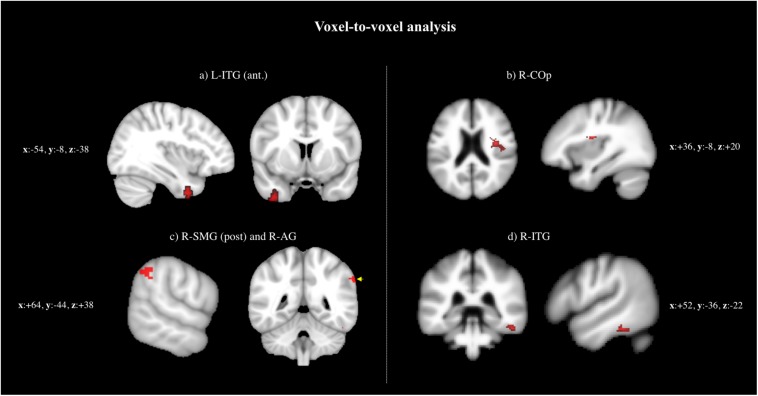
Voxel-to-voxel analysis: regions demonstrated a difference in peak BOLD signal between the four groups (i.e., AD, PD, FTD, and HC): **(a)** L-ITG (ant.), **(b)** RCOp, **(c)** R-SMG (post.) and R-AG, and **(d)** R-ITG.

The following regions demonstrated a difference in peak BOLD signal between the four groups (i.e., AD, PD, FTD, and HC): (a) left ITG, anterior division (L-ITG, ant), (b) right central opercular cortex (R-COp), (c) right supramarginal gyrus, posterior division (R-SMG, post), and right angular gyrus (R-AG), and (d) right ITG (R-ITG) (Supplementary Table 1 and Figure 1).

### ROI-to-Voxel Analysis

The seed-to-voxel analysis exhibited group differences for each seed extracted for voxel-to-voxel analysis (Supplementary Tables 2A–D, 3A–D). The Fisher *Z*-transformation score for each group is reported in Supplementary Tables 3A–D.

**FIGURE 2:**
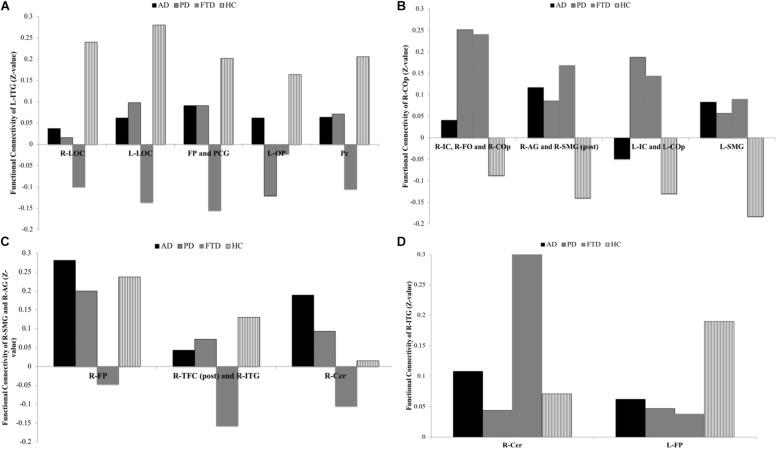
ROI-to-voxel analysis, group differences for each seed extracted for voxel-to-voxel analysis: **(A)** Group differences for functional connectivity of L-ITG, **(B)** group differences for functional connectivity of R-COp, **(C)** group differences for functional connectivity of R-SMG and R-AG, and **(D)** group differences for functional connectivity of R-ITG.

(a) L-ITG (ant) ROI (Supplementary Table 3A and Figure 2A): In the AD, PD, and FTD groups, a decreased functional connectivity was found between the L-ITG (ant) and the right and left lateral occipital cortex (R-LOC and L-LOC) compared to HC. Upon further examining this relationship, the PD and AD groups exhibited almost no functional connectivity, whereas the FTD group demonstrated negative connectivity between the two regions compared with positive connectivity in HC. Furthermore, the FTD group demonstrated decreased (negative) connectivity of L-ITG (ant) with the bilateral FP and paracingulate gyrus (PCG) cluster, and precuneus cortex cluster (Pc), compared to HC. The AD and the PD groups displayed almost no connectivity between the L-ITG (ant), and bilateral FP and Pc; however, this is not significantly different from HC.
(b) R-COp ROI (Supplementary Table 3B and Figure 2B): The functional connectivity of the R-COp was significantly different between HC and the three neurodegenerative groups for several GM regions. Firstly, one of the clusters, which consisted of the right insular cortex (R-IC), right frontal operculum cortex (R-FO), and R-COp, showed positive functional connectivity for PD and FTD, whereas this relationship was absent in AD and HC. Next, three significant clusters (1) R-AG and R-SMG (post), (2) L-IC and L-Cop, and (3) L-SMG were negatively functionally connected with the R-COp in HC, whereas this relationship was not observed in the three neurodegenerative groups.
(c) R-SMG (post) and R-AG ROI (Supplementary Table 3C and Figure 2C): The R-SMG (post) and R-AG also showed distinct patterns of functional connectivity between the four groups. The functional connectivity with the R-FP was positive in the HC, AD, and PD group, whereas this association was absent in the FTD group. In addition, the FTD group displayed negative functional connectivity with the right temporal fusiform cortex, posterior division (R-TFC, post), and R-ITG, whereas there was positive functional connectivity in HC, and no connectivity in AD and PD. Lastly, a significant difference in connectivity was observed between FTD, and AD and PD, with the right cerebellum (R-Cer).
(d) R-ITG (post) ROI (Supplementary Table 3D and Figure 2D): For the R-ITG (post) ROI, the FTD group displayed significantly increased connectivity with the R-Cer, compared to AD. The PD group, on the other hand, displayed no functional connectivity with the L-FP, compared to HC.

### Association Between Functional Connectivity and Social Cognition Measures

In order to determine whether there was a relationship between differences in functional connectivity in the four groups and the social cognition measures, we only examined regions relevant to social cognition. The following regions are known to be involved in social cognition: (a) FP, (b) temporal lobe, and (c) insular cortex (Lamm and Singer, 2010; Elamin et al., 2012). Therefore, the following functionally connected regions from the ROI-to-voxel analysis were examined in all participants combined: (a) L-ITG (ant) with bilateral FP, PCG, (b) R-COp with R-IC, R-FO, R-COp, (c) R-COp with L-IC and L-COp, (d) R-SMG (post), R-AG with R-FP, (e) R-SMG (post), R-AG with R-TFC (post), R-ITG, and (f) R-ITG (post) and L-FP.

**FIGURE 3:**
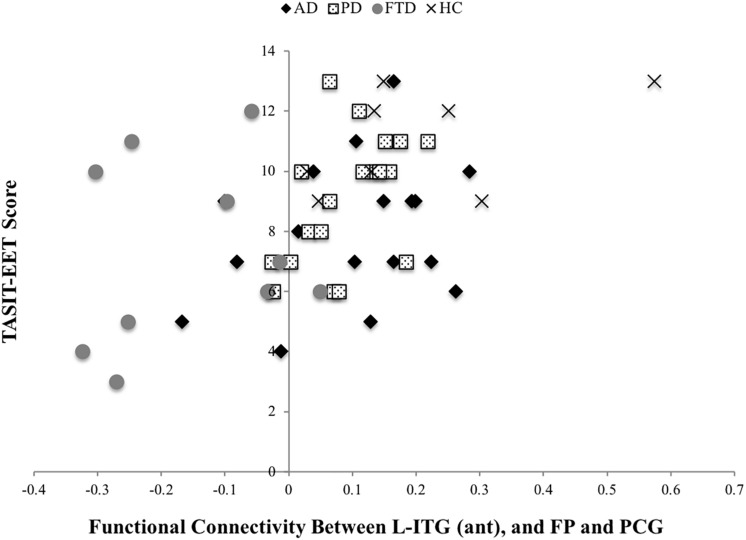
TASIT-EET association with connectivity between L-ITG (ant) and bilateral FP, PCG.

The BOLD co-activation of the L-ITG (ant) with bilateral FP, PCG was positively associated with IRI-PT (*r* = 0.38, *p* = 0.007), SNQ total (*r* = 0.37, *p* = 0.009), and TASIT-EET (*r* = 0.47, *p* < 0.001). There was also a positive correlation of functional connectivity of the R-SMG (post), R-AG, and R-FP with IRI-EC (*r* = 0.32, *p* = 0.029), IRI-PT (*r* = 0.43, *p* = 0.002), RSMS-EX (*r* = 0.42, *p* = 0.003), RSMS-SP (*r* = 0.35, *p* = 0.014), and a trend for TASIT-EET (*r* = 0.27 *p* = 0.06). Lastly, the IRI-PT (*r* = 0.34, *p* = 0.017) and TASIT-EET (*r* = 0.42, *p* = 0.003) were also positively associated with the functional connectivity of R-SMG (post), R-AG with R-TFC, and R-ITG. However, only the TASIT-EET association with connectivity between L-ITG (ant) and bilateral FP, PCG withstood correction for multiple comparisons at *p* < 0.0012 (Figure 3).

### Uncinate Fasciculus (UF) Structural Integrity Analysis

Since the FP and the ITG (anterior division) are structurally connected by the UF (Von Der Heide et al., 2013), we used one-way ANOVA, followed by Dunnett’s T3 *post hoc* analysis to examine the structural integrity of this white matter tract across the four groups (Table 2).

**TABLE 2:** DTI parameters [mean (SD)] for the left and right uncinate fasciculus.

	**AD**	**PD**	**FTD**	**HC**	***p*-value**
Right UF FA	0.32 (0.03)	0.34 (0.03)	0.31 (0.06)	0.34 (0.04)	0.287
Right UF AxD^†^	1.31 (0.10)	1.34 (0.10)	1.45 (0.20)	1.28 (0.10)	0.009
Right UF RD^†^	0.80 (0.07)	0.80 (0.07)	0.92 (0.03)	0.75 (0.05)	0.029
Right UF MD^†^	0.97 (0.07)	0.98 (0.07)	1.00 (0.24)	0.93 (0.05)	0.018
Left UF FA	0.33 (0.04)	0.31 (0.04)^d^	0.28 (0.04)^c^	0.31 (0.04)	0.043
Left UF AxD^†^	1.13 (0.10)	1.30 (0.10)	1.43 (0.10)^a^	1.21 (0.10)^d^	<0.001
Left UF RD^†^	0.81 (0.10)	0.79 (0.10)^d^	0.96 (0.10)^a,c^	0.74 (0.10)^d^	0.001
Left UF MD^†^	0.97 (0.10)	0.96 (0.08)^d^	1.12 (0.15)^a,c^	0.90 (0.07)^d^	<0.001

*^*a*^*p* < 0.05 compared to HC; ^*b*^*p* < 0.05 compared to AD; ^*c*^*p* < 0.05 compared to PD; ^*d*^*p* < 0.05 compared to FTD. ^†^Data presented as mm^2^ s^–1^ 10^–3^.*

**FIGURE 4:**
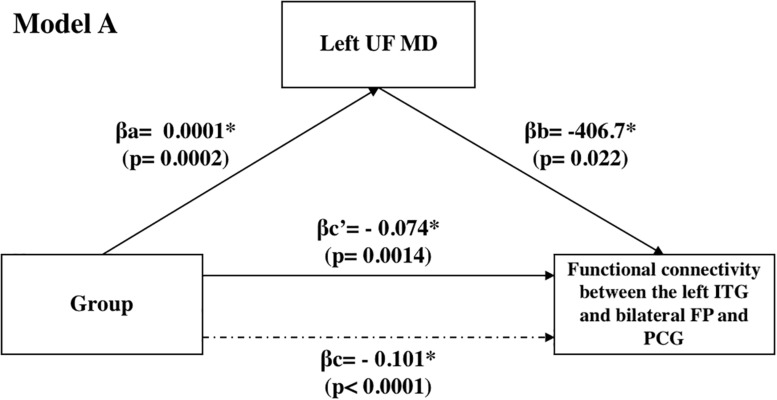
Mediation analyses: effect of the UF on the observed effects of “group” on the functional connectivity between the L-ITG (ant), and bilateral FP and PCG.

We also carried out mediation analyses to explore the potential mediation effect of the UF on the observed effects of “group” on the functional connectivity between the L-ITG (ant), and bilateral FP and PCG. These analyses revealed that the effect of “group” on functional connectivity was partially mediated through the structural connectivity of the left UF [Table 3 (Model A) and Figure 4]. However, the mediation ratio (i.e., ratio of indirect to total effect) was only 26.3% (Table 3), which showed that the effect of “group” on the functional connectivity between the L-ITG and bilateral FP and PCG was beyond the effects of structural connectivity of the left UF alone. The right UF MD showed no significant mediating effect on the relationship between the “group” and functional connectivity of right ITG and FP (Table 3, Model B).

**TABLE 3:** Parameters of mediation analyses.

	**Whole model**				**Paths**				
			
	***R*^2^**	***F***	***p***^∗^		**β/effect**	**SE**	***t***	***p***^∗^	**95% CI**
Model A									
	0.421	11.4	<0.0001	Total effect (path c)	−0.101	0.020	−5.1	<0.0001	−0.14 to −0.06
				Direct effect (path c′)	−0.074	0.022	−3.4	0.0014	−0.12 to −0.03
				Indirect effect	−0.027	0.014			−0.06 to −0.002
				^†^Ratio of indirect to total effect	0.263	0.152			0.02–0.62
				Path a	0.0001	0.0000	4.1	0.0002	0.0000–0.0001
				Path b	−406.7	171.4	−2.4	0.022	−751.7 to −61.7
Model B									
	0.107	1.87	0.148	Total effect (path c)	−0.011	0.019	−0.58	0.565	−0.05 to 0.03
				Direct effect (path c′)	−0.01	0.020	−0.484	0.631	−0.05 to 0.03
				Indirect effect	−0.001	0.0062			−0.02 to 0.001
				^†^Ratio of indirect to total effect	0.210	0.125			0.02–0.55
				Path a	0.0000	0.0000	2.7	0.0089	0.0000–0.0001
				Path b	−22.8	165.2	−0.14	0.891	−355.2 to 309.6

*^†^Mediation ratio. ^∗^In all mediation analyses, age considered as a covariate. β, beta coefficient; SE, standard error; CI, confidence interval; path c, total effect of the independent variable (i.e., group) on the dependent variable (i.e., functional connectivity between the left ITG and bilateral FP and PCG); path c′, direct effect of the independent variable on the dependent variable; path a, effect of the independent variable on the mediator (i.e., left uncinate fasciculus mean diffusivity); path b, effect of the mediator on the dependent variable.*

## Discussion

Our results reveal alterations in functional connectivity in patients with AD, PD, and FTD compared to HC. There was a difference in BOLD signal activation in the L-ITG (ant), R-COp, R-SMG and R-AG, and R-ITG (post) among the four groups. These areas displayed altered connectivity with other regions in the neurodegenerative disease groups. While the HC group displayed positive functional connectivity between the L-ITG (ant) and all its clusters, the AD group exhibited no functional connectivity between this area and most of the regions and decreased functional connectivity between the R-COp, and the R-AG and R-SMG (post) in the AD group. The functional connectivity between the R-ITG and bilateral LOC was also decreased in PD but in a distinct pattern from the AD group. In the PD group, there was increased connectivity between the R-COp, and (a) the R-IC, R-FO, and R-COp, as well as (b) the L-IC and L-COp, compared to the HC and the AD group. Furthermore, there was no connectivity between the R-ITG (post) and the L-FP, in contrast to the HC and AD groups.

Most of the previous studies identified neural substrates underlying emotional empathy by comparing stimuli with different emotional intensities (Breiter et al., 1996; Morris et al., 1996; Phillips et al., 1997; Sprengelmeyer et al., 1998; Blair et al., 1999), by comparing the perception of emotions and the observation of others experiencing the same emotions (Wicker et al., 2003; Jabbi et al., 2007; Jabbi and Keysers, 2008), or by comparing the perception of emotions with the imitation of the same emotions (Carr et al., 2003). While these studies found neural activity in brain areas such as the ACC, anterior insula, superior temporal cortex, amygdala, and inferior frontal cortex (Breiter et al., 1996; Morris et al., 1996; Phillips et al., 1997; Sprengelmeyer et al., 1998; Blair et al., 1999; Carr et al., 2003; Wicker et al., 2003; Hofmann, 2006; Jabbi et al., 2007; Shdo et al., 2018), the designs employed in the previous work did not allow to isolate intentionally controlled processes from automatically generated processes of empathy. In addition, although a number of studies investigated the modulation of “empathy for pain” by cognitive mechanisms (Lamm et al., 2007; Hein and Singer, 2008) or experience to painful practices (Cheng et al., 2007), the neuronal basis underlying the cognitive modulation of “emotional empathy” has, to our knowledge, not been investigated so far.

### Social Cognition in Neurodegenerative Groups

All three neurodegenerative groups scored significantly lower on perspective-taking, a measure of empathy, compared to HC (Davis, 1983) and this was positively associated with the functional connectivity of the L-ITG (ant) with bilateral FP and PCG, as well as the connectivity between R-SMG, R-AG cluster with the (a) R-FP, and (b) R-TFC (post), R-ITG. These areas have been previously implicated in empathy in functional MRI studies (Breiter et al., 1996; Morris et al., 1996; Phillips et al., 1997; Sprengelmeyer et al., 1998; Blair et al., 1999; Carr et al., 2003; Hofmann, 2006; Jabbi et al., 2007; Shdo et al., 2018). Compared to the HC, the FTD group performed worse on the SNQ total, a measure of social norm knowledge. The SNQ total showed a positive association with functional connectivity between the L-ITG (ant), and bilateral FP and PCG. The anterior temporal lobe volume has been related to social norms performance on SNQ in bvFTD (Panchal et al., 2016) but our data show that the functional connectivity of the ITG with FP and PCG is related to the SNQ across all the neurodegenerative diseases so although the anterior temporal lobe is not usually atrophied in AD or PD, altered functional connectivity in temporal and frontal lobes can also contribute to social norms deficits.

The TASIT-EET score also exhibited a positive correlation with the functional connectivity between the R-SMG (post) and R-AG cluster with the R-TFC (post) and ITG. Lastly, although the IRI-EC, RSMS-EX, and RSMS-SP did not withstand correction for multiple comparisons, they were positively associated with the functional connectivity of R-SMG, R-AG with the R-FP. The alterations observed in the functional connectivity of these areas in the patients can explain some of the impairments in these social cognition tasks.

Overall, the findings of this study suggest positive functional connectivity between the L-ITG and bilateral FP and PCG is related to increased ability to take on others’ point of view. Perspective-taking requires one to deliberately suppress self-perception in order to reflect on others’ point of view and represents cognitive empathy (Ruby and Decety, 2003). Loss of cognitive and affective empathy is prominent in bvFTD and cognitive empathy deficits are also reported in individuals with AD (Dermody et al., 2016). In both groups, distinct patterns of atrophy are associated with cognitive empathy impairment. In the AD group, this is primarily related to the left temporoparietal atrophy, whereas bilateral frontoinsular, temporal, parietal, and occipital atrophy is associated with loss of cognitive empathy in the bvFTD group (Dermody et al., 2016). Furthermore, the FP plays a key role in inhibiting self-perception when assessing situations from a third person’s viewpoint (Ruby and Decety, 2003). Therefore, the loss of cognitive empathy in the neurodegenerative groups in the current study can be attributed to loss of functional connectivity between the L-ITG and bilateral FP and PCG.

### Distinct Pattern of Functional Connectivity in FTD

The most distinct pattern of functional connectivity was observed in the FTD group. Our results reveal that the FTD group showed negative connectivity between the R-ITG (ant), and (a) R-LOC; (b) L-LOC; (c) bilateral FP and PCG; and (d) Pc cortex, compared to HC and was also significantly different from the AD and PD groups. Functional connectivity between the R-COp cortex and the R-IC, R-FO, and R-COp cortex was present in FTD, whereas this association was absent in the HC and AD group. The R-COp cortex functional connectivity was negative with the (a) R-AG and R-SMG (post) and (b) L-IC and L-COp in HC, while it was positive in the FTD group. Moreover, R-COp cortex connectivity with L-SMG was absent in FTD but positive in HC. The R-SMG (post) and R-AG seed exhibited no connectivity with the R-FP in the FTD group, compared to AD, PD, and HC. Moreover, the R-SMG (post) and AG displayed negative connectivity with the R-TFC (post) and R-ITG, whereas the HC group showed positive connectivity. There was also negative functional connectivity of the R-SMG (post) and R-AG with the R-Cer, and this association was absent in the HC group. Lastly, the R-ITG (post) was functionally connected to the R-Cer. This pattern was not observed in the HC group and was also significantly different from the AD and PD groups.

Functional connectivity between the R-FP, and R-SMG and R-AG was absent in the FTD group, and this also correlates with decreased cognitive empathy and impairment in emotion detection. The R-SMG is involved in egocentricity bias, where one projects one’s own beliefs onto others in social scenarios (Silani et al., 2013). Empathy also requires social perception, which entails non-verbal cues such as body language, facial expression, and eye-gazing, as well as higher mental processes (ToM) (Pelphrey et al., 2004). Social perception has been attributed to the R-SMG, R-AG, superior parietal lobe, and parahippocampal gyri (Lawrence et al., 2006). As the FP inhibits self-perception and the R-SMG is a key component of egocentricity bias and social perception (including R-AG), we hypothesize that this functional synchronization between the three regions may allow individuals to inhibit (FP) their egocentricity bias (R-SMG) and allow them to take on others’ perspective by mediating social perception cues. Lastly, the positive functional association between R-SMG and R-AG with R-TFC (post) and R-ITG observed in the HC group was absent in the AD and PD groups and was negative in FTD. Its association with cognitive empathy suggests that this loss of connectivity in individuals with FTD may be responsible for their inability to appreciate social perception cues and the negative association with the R-TFC (post) and R-ITG may be responsible for FTD patient’s inability to process emotional facial cues (right posterior fusiform cortex) (Geday et al., 2003) and therefore they are impaired in detecting others’ emotions. Hence, we postulate that individuals who display altered functional connectivity pattern in these regions may have trouble dissociating from self-referential thinking. As a result, they are unable to empathize and are inaccurate at detecting emotions in others.

Overall, the findings of this study suggest that alteration in functional connectivity of the L-ITG, and R-SMG and R-AG with social cognition-relevant regions (i.e., FP, temporal lobe, and insular cortex) is associated with decreased cognitive empathy and emotion detection impairment in neurodegenerative diseases, particularly FTD but also in AD and PD. Changes in social cognition have been associated with caregiver burden and depression (Martinez et al., 2018) especially when unrecognized. Our findings could provide a basis for using resting state functional connectivity as a biomarker of deficits in social cognition. It may also be an early sign of disease and so should be evaluated in the early stages. This altered connectivity may be amenable to interventions so functional connectivity may also prove useful as an outcome measure in interventional studies.

## Data Availability Statement

The datasets generated for this study are available on request to the corresponding author.

## Ethics Statement

The studies involving human participants were reviewed and approved by the University Health Network Research Ethics Board. The patients/participants provided their written informed consent to participate in this study.

## Author Contributions

NM and FT carried out the acquisition of data, analyzed and interpreted the data, and drafted the manuscript for intellectual content. CA, BV, and KM performed a major role in the acquisition of data. DT-W, RK, SF, AL, AV, and CM interpreted the data and revised the manuscript for intellectual content. MT performed a major role in the acquisition of data, interpreted the data, drafted the manuscript for intellectual content, and revised the manuscript for intellectual content.

## Conflict of Interest

The authors declare that the research was conducted in the absence of any commercial or financial relationships that could be construed as a potential conflict of interest.

## Footnotes

1 www.nitrc.org/projects/conn

2 https://fsl.fmrib.ox.ac.uk/fsl/fslwiki/FDT
