# Supplementary material for: Association Between Social Cognition Changes and Resting State Functional Connectivity in Frontotemporal Dementia, Alzheimer’s Disease, Parkinson’s Disease, and Healthy Controls
Source: Front Neurosci. 2019 Nov 22;13:1259. doi: 10.3389/fnins.2019.01259 (PMC6883726; doi:10.3389/fnins.2019.01259)
Supplement: Supplementary file 1 [file Table_1.DOCX]

Table 1. Voxel-to-voxel analysis for difference in BOLD activation between AD, PD, FTD and HC.

| Region | Center coordinates (MNI space) | | | Cluster p-FWE | Size (voxels) |
| --- | --- | --- | --- | --- | --- |
|  | x | y | z |  |  |
| L-ITG (ant) | -54 | -08 | -38 | < .001 | 205 |
| R-COp | +36 | -08 | +20 | < .001 | 84 |
| R-SMG and R-AG | +64 | -44 | +38 | < .001 | 64 |
| R-ITG (post) | +52 | -36 | -22 | < .001 | 54 |

*Only clusters with cluster-size above 50 voxels are reported

*L-ITG (ant) – left inferior temporal gyrus (anterior division); R-COp – right central opercular cortex; R-SMG (post) and R-AG

- right supramarginal (posterior division) and angular gyrus; R-ITG (post) - right inferior temporal gyrus (posterior division)

Table 2. ROI-to-voxel analysis

2A. ROI: Left inferior temporal gyrus (anterior division)

| Region | Center coordinates (MNI space) | | | Cluster p-FDR | Size (voxels) |
| --- | --- | --- | --- | --- | --- |
|  | x | y | z |  |  |
| R-LOC | +44 | -76 | +36 | < .001 | 2748 |
| L-LOC | -36 | -76 | +38 | < .001 | 1305 |
| FP and PCG | -08 | +54 | +16 | < .001 | 1002 |
| L-OP | -24 | -96 | +20 | .0002 | 641 |
| Pc | -02 | -64 | +28 | .03 | 231 |

R-LOC – right lateral occipital cortex; L-LOC – left lateral occipital cortex; FP and PCG - bilateral frontal pole and paracingulate gyrus; L-OP – left occipital pole; Pc – precuneous cortex

2B. ROI: Right central opercular cortex

| Region | Center coordinates (MNI space) | | | Cluster p-FDR | Size (voxels) |
| --- | --- | --- | --- | --- | --- |
|  | x | y | z |  |  |
| R-IC, R-FO and R-COp | +46 | +10 | +10 | < .001 | 1042 |
| R-AG and R-SMG (post) | +46 | -48 | +40 | < .001 | 982 |
| L-IC and L-COp | -32 | +08 | +04 | < .001 | 760 |
| L-SMG | -60 | -40 | +34 | .02 | 229 |

R-IC, R-FO and R-COp - right insular cortex, frontal and central operculum cortex; R-AG and R-SMG (post) - right angular gyrus and supramarginal gyrus (posterior division); L-IC and L-COp - left insular cortex and left central operculum cortex; L-SMG - left supramarginal gyrus

2C. ROI: Right supramarginal (posterior division) and right angular gyrus

| Region | Center coordinates (MNI space) | | | Cluster p-FDR | Size (voxels) |
| --- | --- | --- | --- | --- | --- |
|  | x | y | z |  |  |
| R-FP | +48 | +48 | +28 | .002 | 520 |
| R-TFC (post) and R-ITG | +34 | -20 | -36 | .005 | 392 |
| R-Cer | -04 | -70 | -58 | .01 | 291 |

R-FP – right frontal pole; R-TFC (post) and R-ITG - right temporal fusiform cortex (posterior division) and right inferior temporal gyrus; R-Cer – right cerebellum

2D. ROI: Right inferior temporal gyrus (posterior division)

| Region | Center coordinates (MNI space) | | | Cluster p-FDR | Size (voxels) |
| --- | --- | --- | --- | --- | --- |
|  | x | y | z |  |  |
| R-Cer | +34 | -56 | -24 | < .001 | 1103 |
| L-FP | -50 | +46 | -12 | .04 | 257 |

R-Cer – right cerebellum; L-FP – left frontal pole

Table 3. ROI-to-voxel analysis: Difference in functional connectivity between AD, PD, FTD and HC.

Table 3A. ROI: Left inferior temporal gyrus (anterior division)

| ROIs | AD (z-value) | PD (z-value) | FTD (z-value) | HC (z-value) | Group differences* |
| --- | --- | --- | --- | --- | --- |
| R-LOC | .037 + .01 | -.064 + .08 | -.1 + .01 | .24 + .07 | HC > AD, PD, FTD  AD > PD, FTD |
| L-LOC | .062 + .14 | .098 + .13 | -.136 + .09 | .28 + .1 | HC > AD, PD, FTD  FTD < AD, PD |
| FP and PCG | .091 + .13 | .091 + .07 | -.155 + .14 | .202 + .18 | FTD < HC, AD, PD |
| L-OP | .062 + .11 | -.121 + .11 | -.023 + .11 | .164 + .13 | HC > PD, FTD  AD > PD |
| Pc | .064 + .11 | .071 + .14 | -.105 + .13 | .206 + .15 | FTD < HC, AD, PD |

*Significant group differences upon correcting for multiple comparisons

Table 3B. Right central opercular cortex

| ROIs | AD (z-value) | PD (z-value) | FTD (z-value) | HC (z-value) | Group differences* |
| --- | --- | --- | --- | --- | --- |
| R-IC, R-FO and R-COp | .041 + .15 | .252 + .15 | .241 + .16 | -.088 + .06 | HC < AD, PD, FTD  AD < PD, FTD |
| R-AG and R-SMG (post) | .117 + .11 | .086 + .09 | .169 + .1 | -.14 + .1 | HC < AD, PD, FTD |
| L-IC and L-COp | -.049 + .16 | .188 + .12 | .144 + .12 | -.13 + .1 | HC < PD, FTD  AD < PD, FTD |
| L-SMG | .083 + .13 | .057 + .11 | .09 + .12 | -.183 + .09 | HC < AD, PD, FTD |

Table 3C. ROI: Right supramarginal (posterior division) and angular gyrus

| ROIs | AD (z-value) | PD (z-value) | FTD (z-value) | HC (z-value) | Group differences* |
| --- | --- | --- | --- | --- | --- |
| R-FP | .281 + .13 | .2 + .12 | -.047 + .13 | .237 + .08 | FTD < AD, PD, HC |
| R-TFC (post) and R-ITG | .043 + .11 | .072 + .11 | -.157 + .09 | .13 + .08 | FTD < AD, PD, HC |
| R-Cer | .189 + .12 | .093 + .13 | -.105 + .14 | .015 + .18 | FTD < AD, PD |

Table 3D. ROI: Right inferior temporal gyrus (posterior division)

| ROIs | AD (z-value) | PD (z-value) | FTD (z-value) | HC (z-value) | Group differences* |
| --- | --- | --- | --- | --- | --- |
| R-Cer | .108 + .15 | .044 + .14 | .306 + .14 | -.039 + .11 | FTD > AD, PD, HC |
| L-FP | .062 + .08 | -.063 + .11 | .038 + .17 | .19 + .13 | PD < AD, HC |

<https://www.nitrc.org/forum/forum.php?thread_id=3317&forum_id=1144>
