# Supplementary material for: Association Between Social Cognition Changes and Resting State Functional Connectivity in Frontotemporal Dementia, Alzheimer’s Disease, Parkinson’s Disease, and Healthy Controls
Source: Front Neurosci. 2019 Nov 22;13:1259. doi: 10.3389/fnins.2019.01259 (PMC6883726; doi:10.3389/fnins.2019.01259)
Supplement: Supplementary file 2 [file Table_2.DOCX]

Supplemental

**TorCA:** A standardized cognitive assessment, which measures function in the following subdomains: attention, memory, language, visuospatial function, executive function and praxis(Freedman *et al.*, 2018)

**CDR:** A global assessment scale to measure dementia severity stage(Morris, 1993). The CDR sum of boxes (CDR-SOB), which is used to stage dementia study, is examined for the current study.

**TASIT-EET:** A multimodal emotion detection task comprising 14 short (~20 second) video clips to assess subjects’ ability to identify emotions with ecologically valid, dynamic, multimodal stimuli. At the end of each clip, a display with a list of seven emotions (happiness, surprise, sadness, anger, fear, disgust and neutral) is presented to the participants. Participants are instructed to choose an emotion, which most accurately represents the emotion portrayed by the actor(McDonald *et al.*, 2003).

**BIS/BAS**: To measure behavioural inhibition (sensitivity to punishment) and behavioural activation (sensitivity to rewards) in individuals(Carver and White, 1994). Four behavioural inhibition system (BIS) and behavioural activation system scores are obtained 1)BIS; 2)BAS drive (BAS-D); 3)BAS fun seeking (BAS-FS); and 4)BAS reward responsiveness (BAS-RR). For the purpose of this paper, only the BIS score was examined.

**RSMS:** In order to measure the subjects’ awareness of their own social behavior (as assessed by the informant), we obtained informant’s perspective of the subject’s self-concern and self-focus using the Lennox and Wolfe version of the Revised Self-Monitoring Scale (RSMS) informant-based reports(Lennox and Wolfe, 1984). The RSMS is a 13-item measure of a subject’s sensitivity to the expressive behavior of others, and their ability to monitor their self-presentation. RSMS is often reported by informants and has been validated in neurodegenerative diseases(Hofmann, 2006; Shdo *et al.*, 2018). A RSMS total score and two sub-scores 1)Sensitivity to Socio-emotional Expressiveness Score (EX) and 2)Ability to Modify Self-presentation Score (SP) were obtained.

**IRI:** Empathy was evaluated using the Interpersonal Reactivity Index (IRI), which measures both the cognitive and emotional aspects of empathy(Davis, 1983). Informants ﬁll out the questionnaire describing the subject’s current characteristics. Four sub-scores are obtained 1)Perspective Taking (PT); 2)Fantasy (F); 3)Empathic Concern (EC); and 4)Personal Distress (PeD). For the purpose of this paper, only the PT and EC scores were examined.

**SNQ:** The SNQ(Rankin, 2008) consists of 22 statements and assesses the subject’s ability to assess social boundaries in the mainstream culture of Canada. The subject is presented with statements pertaining to social situations and were instructed to respond yes/no whether it is socially appropriate to carry out those acts.
